# Time pressure reduces misinformation discrimination ability but does not alter response bias

**DOI:** 10.1038/s41598-022-26209-8

**Published:** 2022-12-27

**Authors:** Mubashir Sultan, Alan N. Tump, Michael Geers, Philipp Lorenz-Spreen, Stefan M. Herzog, Ralf H. J. M. Kurvers

**Affiliations:** 1 Max Planck Institute for Human Development, Center for Adaptive Rationality, Berlin, 14195 Germany; 2 Department of Psychology, Humboldt University of Berlin, 12489 Berlin, Germany; 3 Technical University of Berlin, Exzellenzcluster Science of Intelligence, Berlin, 10587 Germany

**Keywords:** Human behaviour, Psychology

## Abstract

Many parts of our social lives are speeding up, a process known as social acceleration. How social acceleration impacts people’s ability to judge the veracity of online news, and ultimately the spread of misinformation, is largely unknown. We examined the effects of accelerated online dynamics, operationalised as time pressure, on online misinformation evaluation. Participants judged the veracity of true and false news headlines with or without time pressure. We used signal detection theory to disentangle the effects of time pressure on discrimination ability and response bias, as well as on four key determinants of misinformation susceptibility: analytical thinking, ideological congruency, motivated reflection, and familiarity. Time pressure reduced participants’ ability to accurately distinguish true from false news (discrimination ability) but did not alter their tendency to classify an item as true or false (response bias). Key drivers of misinformation susceptibility, such as ideological congruency and familiarity, remained influential under time pressure. Our results highlight the dangers of social acceleration online: People are less able to accurately judge the veracity of news online, while prominent drivers of misinformation susceptibility remain present. Interventions aimed at increasing deliberation may thus be fruitful avenues to combat online misinformation.

## Introduction

A growing body of research has started to suggest that various parts of our social lives are speeding up, a phenomenon referred to as social acceleration^1–3^. Social media is no exception here. Analysing content consumption across multiple platforms (e.g., Twitter and Reddit) over the course of a decade, Lorenz-Spreen et al.^2^ found evidence for increasingly faster flows of collective attention on cultural items (e.g., via hashtags on Twitter and comments on Reddit; see also^4–6^). Acceleration has also been observed at the level of the individual, with later-joining Twitter cohorts being more active (e.g., posting more tweets over time) and switching their focus to new topics more quickly^7^. These accelerating online dynamics are likely to be driven by the increasing production and consumption of content: Social media users are consuming ever more content like news in ever shorter time spans^2,3,8^.

The psychological implications of this information overload^9^ are largely unclear, especially with respect to people’s ability to detect misinformation online. With increasing numbers of people sourcing and sharing news on social media^10^, there is growing public and scholarly concern surrounding the digital spread of misinformation^11–14^. Here, we assess the effect of time pressure—an important corollary of accelerating online dynamics—on people’s susceptibility to misinformation. Specifically, we investigated how time pressure influences (a) the ability to distinguish between true and false news (i.e., discrimination ability in the context of misinformation), (b) the tendency to classify an item as true or false (i.e., response bias), and (c) the effects of four potential drivers of susceptibility to misinformation on (a) and (b).

Previous research on the role of decision timing has found strong support for a speed–accuracy trade-off across various contexts^15–17^. Generally, faster responses come at the expense of reduced discrimination ability^15^. This effect has been attributed to various factors, including less systematic information processing and reduced information search under time pressure^18^. It has also been suggested that decision makers may resort to ‘fast and frugal’ heuristics^19^ under time pressure. By focusing on the most informative cues, these heuristics require less information and computation and can produce accurate results; they may thus circumvent some of the detrimental effects of time constraints^19^. In the absence of such valid frugal strategies to detect misinformation, we expect time pressure to have a detrimental impact on the ability to distinguish between true and false news.

Time pressure may also influence people’s response bias—the likelihood of selecting one option over another, all other things being equal. In the context of judging the veracity of information online, a response bias would manifest as a systematic tendency to identify news items as true or false. As the effect of time pressure on response bias may result from an interplay between initial tendencies and potential strategy shifts under time pressure, this effect is less straightforward to predict than the effect on discrimination ability. A possible hint can be taken from a study by Batailler et al.^20^: They found that participants with lower analytical thinking skills had a higher true-news response bias relative to those with higher analytical thinking skills. Time pressure could reduce the time to engage in analytical thinking, which may lead to a similar increase in a true-news response bias. One has to, however, be cautious with equating a lack of time with a lack of reasoning, especially across people with different levels of analytical thinking skills. Furthermore, experimental results on the effect of time pressure on response bias across domains appear to be mixed^21–26^. In light of the above, we do not have an a priori expectation for the effect of time pressure on response bias.

To date, there has been little research into how time pressure impacts the detection of online misinformation. An exception is the study by Bago et al.^27^, which investigated the role of time pressure on misinformation susceptibility. In the first condition, participants made an initial headline-veracity decision under time pressure (with a 7 s response deadline and while undergoing a cognitive load task). They could then revise their decisions without any time constraints or cognitive load. The revised decisions were more accurate, an effect that was driven mainly by participants believing false news less (but not by believing true news more). In a second condition, participants made their decisions without any time constraints or cognitive load; these were also more accurate compared to the responses under time pressure in the first condition. Increased accuracy after deliberation was found for both politically congruent and incongruent news headlines. Our study contributes to this strand of research in two ways: First, we investigated the general impact of time pressure on misinformation susceptibility (i.e., without the additional impact of cognitive load). Second, and more importantly, we adopted a signal detection theory^28^ (SDT) approach, which allowed us to disentangle the effects of time pressure on discrimination ability and response bias, as well as the effects of key determinants of misinformation susceptibility (e.g., ideological congruency) on discrimination ability and response bias.

Batailler et al.^20^ recently applied SDT to study the effect of four key determinants of misinformation susceptibility on discrimination ability and response bias, namely: (a) analytical thinking^29–32^; (b) ideological congruency^33,34^ (also known as partisan bias); (c) motivated reflection^35,36^ (see also^37^); and (d) familiarity^38,39^. They found that higher analytical thinking skills were associated with higher discrimination ability, whereas familiarity (i.e., having seen a news headline before) was associated with lower discrimination ability. In terms of response bias, individuals were more likely to judge news headlines as true (a) when they scored lower on analytical thinking; (b) when the headlines were congruent with their political identity (ideological congruency); and (c) when they were familiar with the headlines. There was no effect of motivated reflection, which refers to the notion that individuals who score higher (lower) on analytical thinking are more likely to accept (dismiss) politically-congruent news and to dismiss (accept) politically-incongruent news. In line with this conceptualisation of the congruency effect (b), we also conceptualise motivated reflection to affect response bias and not discrimination ability.

Adopting an SDT approach to understand the effects of accelerating information consumption on judging the veracity of misinformation, we examined how time pressure influences discrimination ability and response bias, as well as the influence of four key determinants of misinformation susceptibility on discrimination ability and response bias. To this end, we presented individuals with a mix of 16 true and 16 false news headlines, formatted as they would appear on a social media feed (e.g., Facebook). Half of the true (false) headlines were Republican-leaning and half were Democrat-leaning. Across two treatments, with and without time pressure, participants were asked to judge the veracity of each headline (whether it was true or false), to rate their confidence in their choice, and to state whether they were familiar with the headline. In the time-pressure treatment, participants had to respond to the veracity question within 6 s, a time interval informed by a pilot study (see “Methods”). Given previous research on the speed–accuracy trade-off, we predicted that time pressure would reduce discrimination ability. As results on the effect of time pressure on response bias are mixed, we had no a priori hypothesis on this relationship. We, likewise, had no a priori hypotheses on whether the effects of the four determinants of misinformation susceptibility (i.e., analytical thinking, ideological congruency, motivated reflection, and familiarity) on discrimination ability and response bias would change under time pressure.

## Methods

### Participants

For the main experiment, we recruited 837 participants online via Prolific Academic (www.prolific.co), aiming for an equal split between the control and time-pressure treatment. The study by Bago et al.^27^ was used as an indication for the sample size (see also Supplementary Fig. S1 and Table S1 for a post-hoc power analysis; e.g.^40^). Only participants from the United States, who had a Prolific approval rating greater than 90%, who were either male or female, and who were either Republican or Democrat were allowed to participate. We pre-screened on gender and political identification to ensure a balanced sample. In addition, the following exclusion criteria were applied: whenever participants submitted the experiment twice, only the first attempt was included in the analyses (*n* = 9); missing data on political identification and/or the Cognitive Reflection Test (*n* = 31; two participants who had one response out of four missing on the latter were kept in the analyses); failing at least two (of the six) attention checks (*n* = 30); always giving the same response for the accuracy and/or confidence question (*n* = 7). In total, 77 individuals were excluded from the analyses, resulting in a final sample size of $N=$ 760 participants (*N*_Control_ = 382, *N*_Time Pressure_ = 378; *M*_age_ = 34.5 years, *SD* = 12.2, range = 18–78). Participants received a baseline payment of £2.50 for participation ($\sim$20 min), and £0.05 for every correctly judged news headline (*N*_headlines_ = 32; *M*_bonus_ = £1.14, SD = £0.2, range = £.05–1.6). All procedures were approved by the Institutional Review Board of the Max Planck Institute for Human Development (i2020-06), were conducted in accordance with relevant guidelines and regulations, and participants provided informed consent before starting the experiment.

### Headline selection

We selected a sample of news headlines from a previously pretested bank of 225 headlines by Pennycook et al.^41^, comprising both factually accurate (taken from mainstream sources) and inaccurate (as determined by third-party fact-checking websites such as Snopes.com) headlines. The headlines were presented in Facebook format and consisted of an image, a headline, a byline, and a source. From this bank, we first removed headlines for which the large majority of the original veracity judgements were either correct or incorrect, that is, by removing headlines with accuracy values greater (lesser) than 1.5 times the interquartile range (IQR) above (below) the 3rd (1st) quartile. We next removed headlines that were originally judged to be neutral in terms of political valence, that is, between 3.4 and 3.6 on a 7-point partisanship scale (measured using the question: ‘Assuming the above headline is entirely accurate, how favourable would it be to Democrats versus Republicans?’). Of the remaining headlines, we randomly selected 24 headlines from each of the four possible categories (i.e., true, Republican-leaning; true, Democratic-leaning; false, Republican-leaning; false, Democratic-leaning), resulting in a total of 96 headlines.

We reassessed these headlines in a subsequent test (*N*_participants_ = 150) to determine whether their political valence had changed since the original study by Pennycook et al.^41^. We used Spearman’s rank correlation to compare the political valence ratings in our test sample with those in the original sample; the results showed a strong positive association (${r_{S}}$ = 0.83, *p* < 0.001; see Supplementary Fig. S2). Of the 96 remaining headlines, we removed those that were close to the partisan divide in our test sample, using a bigger divide (i.e., excluding headlines with values between 3.2 and 3.8) while still maintaining enough headlines in each category. Finally, we removed headlines that were often judged as familiar (values > 40%). The final selection consisted of 64 headlines (for histogram on political valence, see Supplementary Fig. S3), which were randomly distributed into two balanced sets of 32 headlines (16 true and 16 false). Each category of 16 headlines contained eight Democratic-leaning and eight Republican-leaning headlines.

### News categorisation task

**Figure 1:**
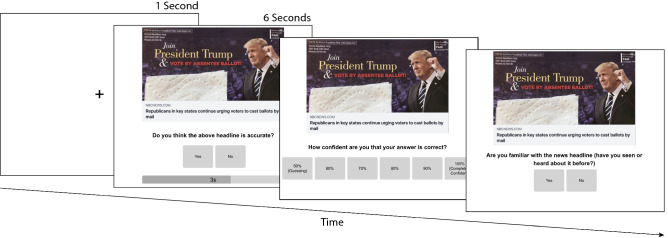
Screenshots from the time-pressure treatment. Each trial started with a fixation cross (1 s), followed by the veracity question that included a 6 s countdown timer, the confidence question, and the familiarity question. The control treatment was identical, except that there was no time limit (and no timer) for the veracity question.

For each news headline, participants answered three questions (see Fig. 1): (1) ‘Do you think the above headline is accurate?’ (‘Yes’ or ‘No’; veracity measure; in the following, we refer to these responses as ‘true’ or ‘false’, respectively); (2) ‘How confident are you that your answer is correct?’ (probability scale ranging from 50% to 100% in increments of 10; data not analysed); and (3) ‘Are you familiar with the news headline (have you seen or heard about it before?’; ‘Yes’ or ‘No’; familiarity measure). The 32 news headlines, along with six attention checks (see screenshots of the experiment on https://osf.io/ag48d/), were randomly presented to participants, who were allowed to take a self-regulated break after the first 16 headlines. Time pressure was manipulated as a between-subjects factor. In the non-time-pressure treatment (i.e., control), participants could take as long as they liked to respond to the veracity question; in the time-pressure treatment, they had to respond within 6 s or forfeit their bonus payment for that trial. The time limit of 6 s was chosen based on data from a pilot study (*N*_participants_ = 55) using the same headlines in a non-time-pressure format. Here, participants had a median response time (RT) of 6.9 s across all trials (see Supplementary Fig. S4 for RT distributions). We thus implemented a slightly shorter time limit in the main study. For the analyses, we removed all trials in the time-pressure treatment with RTs exceeding 6 s (*n* = 208/11883; 2%). In the control treatment, we removed all trials with RTs exceeding 60 s (*n* = 190/12021; 2%). There was no time limit on the confidence or familiarity question in either treatment. Each participant was exposed to only one of the headline sets, and the two test sets were equally distributed across the time treatments, gender, and political identification.

### Demographics and analytical thinking

After the News Categorisation Task, participants answered several demographic questions, most notably including a question on their political identification (Strongly Democratic, Moderately Democratic, Lean Democratic, Lean Republican, Moderately Republican, and Strongly Republican). Participants’ political identification was combined with the political leaning of each news headline (favourable to Democrats or Republicans) to create a measure of ideological congruency (going from strongly incongruent to strongly congruent). For the full list of questions, please see screenshots of the experiment on https://osf.io/ag48d/. Participants next completed the four-item Cognitive Reflection Test^42^ (CRT-2, hereafter referred to as CRT), a less-numeric measure of analytical thinking (sample item: ‘If you’re running a race and you pass the person in second place, what place are you in?’). Upon study completion, participants were debriefed on the veracity of the 32 news headlines, where false headlines were clearly signposted as ‘FALSE’ to reduce belief in misinformation^38^.

### SDT via generalised linear mixed models

We used a Bayesian generalised linear mixed-effects model (GLMM) using the R package brms^43^ (assuming a Bernoulli-distributed response with a probit link function) to implement a mixed-level signal detection model, thus allowing us to differentiate between discrimination ability and response bias (for a detailed overview, see^44–46^). In the GLMM, we used participants’ response to the veracity question (i.e., false or true) as the outcome variable. The predictor variables were headline veracity (i.e., whether the headline was actually false or true); CRT (i.e., proportion of correct responses on the CRT; range 0–1); ideological congruency (on a 6-point Likert scale from strongly incongruent to strongly congruent); motivated reflection (i.e., CRT x ideological congruency); and familiarity (i.e., familiar or unfamiliar). Finally, we modelled random intercepts for participants and news headlines.

The intercept in the regression model reflects the response bias (i.e., the overall likelihood to classify a given headline as true) and the predictors’ coefficients reflect their influence on this response bias. The only exception to this is when the coefficients include headline veracity, which indicates whether the headline was actually true or false. A positive estimate of headline veracity indicates an increased ability to identify true news as true and false news as false (i.e., discrimination ability). The influence of the predictors on discrimination ability is thus inferred via the estimates of their interactions with headline veracity. As we were interested in the impact of time pressure on discrimination ability and response bias, we added the grouping variable time treatment (i.e., control, time pressure) as an interaction term to all predictors. This variable describes the influence of time pressure on response bias; the interaction between headline veracity and time treatment describes the influence of time pressure on discrimination ability.

To aid with model interpretation, all predictors except time treatment and familiarity were mean centered (i.e., value − mean). CRT and ideological congruency were also divided by two standard deviations after mean centering^47^ (for histograms, see Supplementary Figs. S5 and S6). We chose not to center time treatment because this made direct comparison of the control and time treatment easier. We also chose not to center familiarity, as we were predominantly interested in unfamiliar news headlines, hence also the filtering noted in “Headline Selection” (for an analysis with familiarity mean centered, see Supplementary Fig. S7). The parameter estimates were generated by simulating four Markov chain Monte Carlo (MCMC) chains with 10,000 iterations each, discarding the first 5000 as burn-in. We report the mean of the posterior distribution and the 95% credible intervals (CI). Supplementary Table S2 presents an overview of all the model terms to aid interpretation.

## Results

We first present the results for the effects of time pressure on discrimination ability and response bias (i.e., response tendency of classifying a news item as true), followed by the effects of the four determinants of misinformation susceptibility on discrimination ability and response bias. For statistical inference, we relied exclusively on SDT analyses, making it possible to differentiate between discrimination ability and response bias in the same analysis (Fig. 3). To aid interpretation, we also provide descriptive statistics and visualisations (e.g., overall accuracy, accuracy for true [false] headlines).

### Effects of time pressure on discrimination ability and response bias

**Figure 2:**
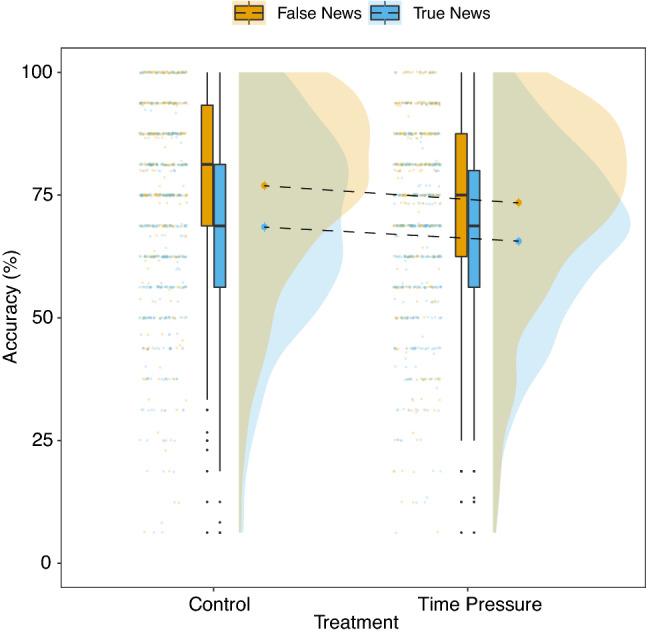
Accuracy (in percentages) for true and false news headlines across the two treatments. The small coloured dots represent the mean accuracy of each individual. The boxplots show the median value and the IQR. The whiskers indicate an additional 1.5 IQR and the small black dots represent outliers. The large coloured dots represent the aggregate mean with standard errors. The density plots describe the distribution of the data. Under time pressure, accuracy was reduced for both true and false headlines (negative slope of dashed lines). In other words, time pressure reduced discrimination ability. Time pressure did not, however, alter the response bias: Accuracy for both true and false news decreased at an equal rate under time pressure.

**Figure 3:**
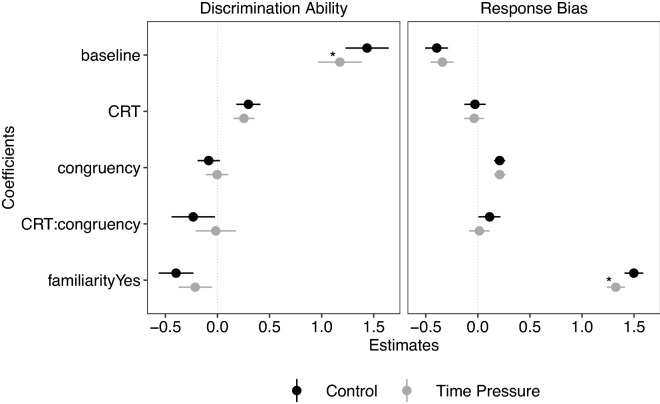
Visualisation of regression coefficients for the control treatment (in black) and the time-pressure treatment (in grey). All results derive from a single SDT analysis using participants’ responses (false news or true news) as the response variable but are shown in two panels to ease interpretation. The left panel shows all estimates for discrimination ability, with more positive (negative) values indicating higher (lower) discrimination ability. The right panel shows all estimates for response bias, with more positive (negative) values indicating a higher (lower) likelihood to judge headlines as true (false). Baseline: Overall estimate of discrimination ability (left panel) and response bias (right panel). CRT = Cognitive Reflection Test. Congruency = ideological congruency. Dots represent the mean; error bars represent the 95% CI of the posterior distribution. Credibly different effects between the control and time pressure treatments are marked with an asterisk (“*”). Note that all effects were mean centered except treatment (reference: control) and familiarity (reference: unfamiliar headlines).

Participants achieved an overall accuracy of 72.7% in the control treatment and 69.6% in the time-pressure treatment (Fig. 2). This was reflected by the estimate of discrimination ability being credibly higher than 0 in both treatments ([${\beta }$_Control_ = 1.44, CI 1.23 to 1.64]; [${\beta }$_Time Pressure_ = 1.17, CI 0.97 to 1.38]; Fig. 3). Discrimination ability was credibly lower in the time-pressure treatment than in the control treatment ([${\beta }$ = $-\,0.26$, CI $-\,0.34$ to $-\,0.18$]), indicating that time pressure reduced participants’ ability to discriminate between true and false news.

Inspection of accuracy for true and false news headlines separately showed that participants in both treatments achieved higher accuracy for false news headlines than for true news headlines (Fig. 2). Participants were, thus, more likely to treat news as false, and this was corroborated by a credibly negative estimate of response bias in both treatments ([${\beta }$_Control_ = $-\,0.39$, CI $-\,0.51$ to $-\,0.29$]; [${\beta }$_Time Pressure_ = $-\,0.34$, CI $-\,0.45$ to $-\,0.23$]; Fig. 3). There was no credible difference between the control treatment and the time-pressure treatment on the response bias ([${\beta }$ = 0.05, CI $-\,0.02$ to 0.13]). In sum, time pressure reduced discrimination ability, but did not alter the—already present—negative response bias.

### Effects of the four determinants of misinformation susceptibility on discrimination ability and response bias

#### Analytical thinking

**Figure 4:**
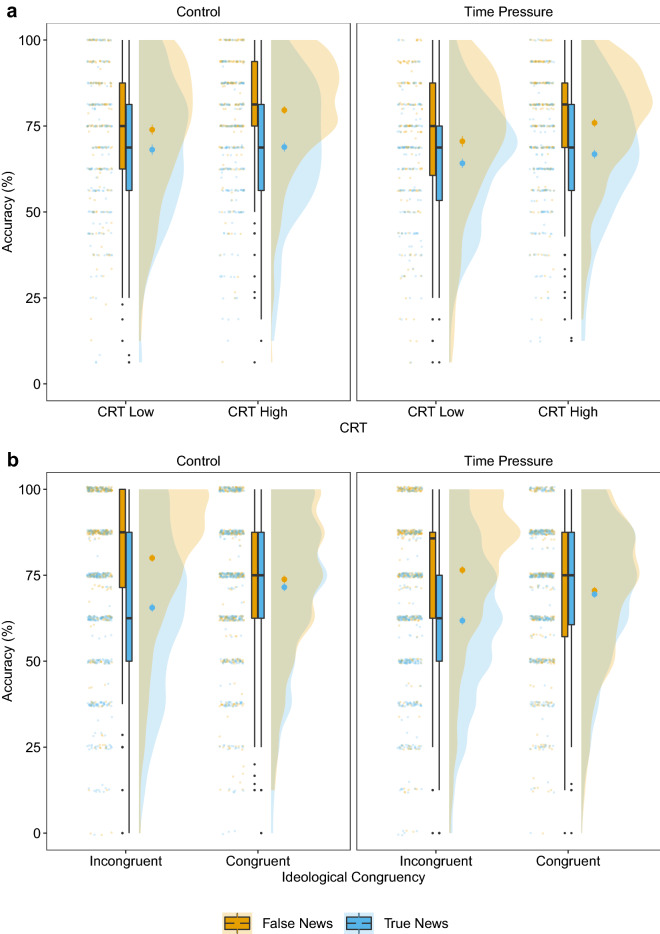
Accuracy for false and true news headlines by CRT scores (**a**) and ideological congruency (**b**) per treatment. CRT scores are shown as High and Low for visualisation purposes but were treated as standardised percentage scores in the analyses. Participants with three or more correct responses were classified as having a High CRT score; participants with two or fewer correct responses as having a Low CRT score. The small coloured dots represent the mean accuracy of each individual. The boxplots show the median value and the IQR. The whiskers indicate an additional 1.5 IQR and the small black dots represent outliers. The large coloured dots represent the aggregate mean with standard errors. The density plots describe the distribution of the data.

Individuals with a higher CRT score had higher overall accuracy in the control treatment (Fig. 4a), reflected in a credibly positive effect of CRT score on discrimination ability ([${\beta }$ = 0.30, CI 0.18–0.41]; Fig. 3). There was no credible difference in the effect of CRT scores on discrimination ability between both treatments ([${\beta }$ = $-\,0.04$, CI $-\,0.19$ to 0.11]). The CRT scores did not have a credible association with response bias in the control treatment ([${\beta }$ = $-\,0.03$, CI $-\,0.13$ to 0.08]; Fig. 3), nor was there a credible difference in CRT scores between treatments ([${\beta }$ = $-\,0.01$, CI $-\,0.15$ to 0.13]). Higher CRT scores were thus associated with increased discrimination ability, and this effect persisted under time pressure; CRT scores were not, however, associated with response bias.

#### Ideological congruency

We found no credible effect of ideological congruency on discrimination ability in the control treatment ([${\beta }$ = $-\,0.08$, CI $-\,0.19$ to 0.02]; Fig. 3), and no credible difference in this effect between the treatments ([${\beta }$ = 0.08, CI $-\,0.07$ to 0.23]). However, we found a positive and credible effect of congruency on response bias in the control treatment ([${\beta }$ = 0.21, CI 0.16–0.26]; Fig. 3). This result implies that participants were more likely to judge a news headline as true (false) if it was congruent (incongruent) with their ideology. Consequently, participants achieved higher accuracy for congruent-true headlines than for incongruent-true headlines (Fig. 4b), but lower accuracy for congruent-false headlines than for incongruent-false headlines (Fig. 4b). The effect of ideological congruency on response bias was not credibly different between the two treatments ([${\beta }$ = 0.00, CI $-\,0.08$ to 0.08]). In sum, ideological congruency was not associated with discrimination ability, but it was associated with an increase in response bias in both treatments. That is, participants were more likely to rate congruent (incongruent) news headlines as true (false; i.e., a partisan bias).

#### Mutual moderation of CRT and ideological congruency

**Figure 5:**
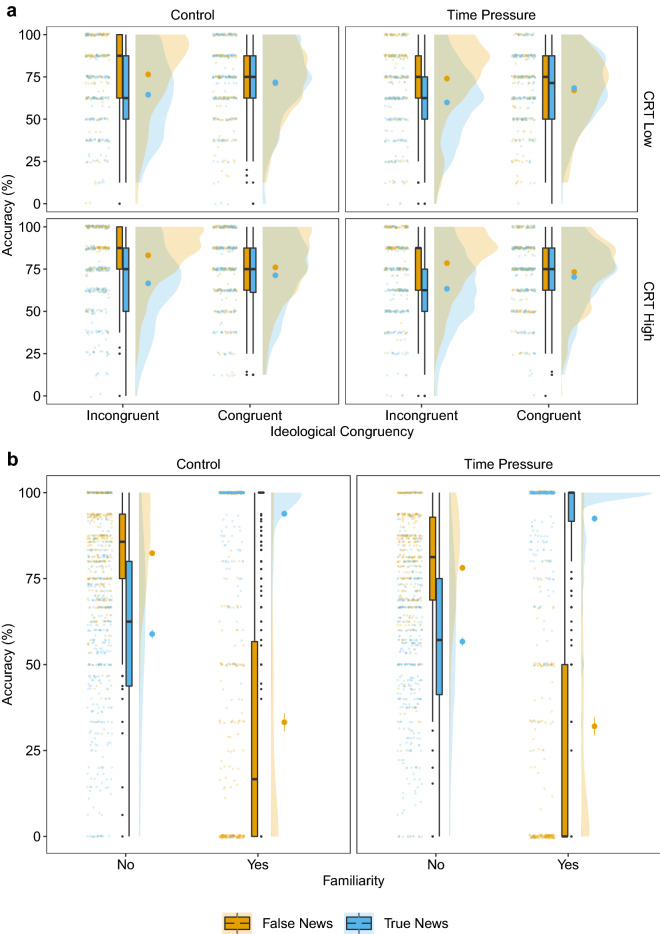
Accuracy for false and true news headlines in both time treatments as a function of CRT and ideological congruency (**a**) and familiarity (**b**). The small coloured dots represent the mean accuracy of each individual. The boxplots show the median value and the IQR. The whiskers indicate an additional 1.5 IQR and the small black dots represent outliers. The large coloured dots represent the aggregate mean with standard errors. The density plots describe the distribution of the data.

The interaction effect of CRT and ideological congruency on discrimination ability was negative and credible in the control treatment ([${\beta }$ = $-\,0.23$, CI $-\,0.44$ to $-\,0.02$]; Fig. 3). As such, the positive effect of higher CRT scores on discrimination ability was smaller for congruent headlines than for incongruent headlines (Fig. 5a). There was no credible difference in this interaction effect on discrimination ability between the two treatments ([${\beta }$ = 0.22, CI $-\,0.07$ to 0.50]). For response bias, the interaction effect of CRT and ideological congruency (i.e., motivated reflection) was credible and positive in the control treatment ([${\beta }$ = 0.11, CI 0.01– 0.22]; Fig. 3): the effect of ideological congruency on response bias (i.e., partisan bias) was likely to be stronger for those with higher CRT scores. This effect was not credibly different between treatments ([${\beta }$ = $-\,0.10$, CI $-\,0.24$ to 0.05]). Please note, however, that the interaction effect of CRT and ideological congruency on both discrimination ability and response bias was no longer credible under time pressure (Fig. 3).

#### Familiarity

The effect of familiarity on discrimination ability was credible and negative in the control treatment ([${\beta }$ = $-\,0.40$, CI $-\,0.56$ to $-\,0.23$]; Fig. 3). Rating a headline as familiar was thus associated with lower discrimination ability. There was no credible difference between the two treatments for the effect of familiarity on discrimination ability ([${\beta }$ = 0.18, CI $-\,0.05$ to 0.41]). We found a strong, positive, and credible effect for familiarity on response bias in the control treatment ([${\beta }$ = 1.50, CI 1.41–1.59]; Fig. 3). Participants were more likely to rate a news headline as true when they indicated that they were familiar with it (i.e., had seen or heard about it before). Consequently, participants achieved higher accuracy scores for familiar-true headlines than for familiar-false headlines but lower accuracy scores for unfamiliar-true headlines than for unfamiliar-false headlines (Fig. 5b). The large effect of familiar news headlines on response bias was, however, credibly weaker under time pressure ([${\beta }$ = $-\,0.17$, CI $-\,0.29$ to $-\,0.05$]). Overall, we found a strong tendency to treat familiar news as true; this tendency decreased slightly under time pressure.

## Discussion

In this study, we investigated the impact of time pressure on people’s ability to judge the veracity of online misinformation in terms of (a) discrimination ability, (b) response bias, and (c) four key determinants of misinformation susceptibility (i.e., analytical thinking, ideological congruency, motivated reflection, and familiarity). We found that time pressure reduced discrimination ability but did not alter the—already present—negative response bias (i.e., general tendency to evaluate news as false). Moreover, the associations observed for the four determinants of misinformation susceptibility were largely stable across treatments, with the exception that the positive effect of familiarity on response bias (i.e., response tendency to treat familiar news as true) was slightly reduced under time pressure. We discuss each of these findings in more detail next.

As predicted, we found that time pressure reduced discrimination ability: Participants under time pressure were less able to distinguish between true and false news. These results corroborate earlier work on the speed–accuracy trade-off^15,18^, and indicate that fast-paced news consumption on social media is likely leading to people misjudging the veracity of not only false news, as seen in the study by Bago and colleagues^27^, but also true news. Like in their paper, we stress that interventions aimed at mitigating misinformation should target this phenomenon and seek to improve veracity judgements by encouraging deliberation. It will also be important to follow up on these findings by examining whether time pressure has a similar effect in the context of news items that have been subject to interventions such as debunking^13,48^.

Our results for the response bias showed that participants had a general tendency to evaluate news headlines as false (i.e., a negative response bias); this effect was similarly strong across the two treatments. From the perspective of the individual decision maker, this response bias could reflect a preference to avoid one type of error over another (i.e., avoiding accepting false news as true more than rejecting true news as false) and/or an overall expectation that false news are more prevalent than true news in our experiment. Note that the ratio of true versus false news we used (1:1) is different from the real world, which typically is thought to contain a much smaller fraction of false news (e.g.^49,50^). A more ecologically valid experiment with a more representative sample could yield a different response bias. It will, thus, be important for future studies to assess whether participants hold such a bias in the real world, are conscious of this response tendency, and whether it translates into (in)accurate beliefs about the news itself.

Findings for the determinants of misinformation susceptibility showed that higher scores on the Cognitive Reflection Test (CRT) resulted in higher discrimination ability, whereas familiarity with a headline resulted in lower discrimination ability. In terms of response bias, ideological congruency resulted in a higher response bias (i.e., a higher likelihood of judging headlines to be true; i.e., partisan bias). This effect was even stronger for participants with higher CRT scores, as indicated by the positive interaction between CRT and ideological congruency (i.e., motivated reflection). Familiarity also increased the response bias. Comparing the control and the time pressure treatment, we found that the effect of motivated reflection was not credible under time pressure, and the association of familiarity on response bias decreased slightly under time pressure (we return to this finding in the next paragraph). Our finding that each determinant of misinformation susceptibility had credible effects on either discrimination ability or response bias (or both in the case of familiarity) highlights that judging the veracity of online news is a complex and multifaceted issue, as conceptualised in several recent reviews^29,33,51,52^. We contribute to this discussion by highlighting possible mechanisms (i.e., discrimination ability and/or response bias) through which the determinants may influence choice behaviour. These results also largely replicate and corroborate those by Batailler et al.^20^ and diverge only regarding the finding for motivated reflection. They did not find such an effect whilst we do, at least in the control—but not time pressure—treatment. Finally, our results show that the effects of most determinants of misinformation persist under time pressure—a nontrivial outcome.

The effect of familiarity, especially on response bias, was by far the strongest, and warrants further discussion. It is reminiscent of the illusory truth effect^38,39,51^, the finding that repeated information is more likely to be perceived as true—due in part to processing fluency. As we did not repeat headlines in our study, we can only speak of a subjective illusory truth effect. That said, it is not essential for information to be repeated to produce an illusory truth effect. As long as the individual has a “subjective experience of ease”^53^ when processing the stimulus, a truth effect may emerge^53–55^. The strong effect of familiarity on response bias was slightly reduced under time pressure, implying a reduced truth effect when time is short. One possible explanation for this is that time pressure may have reduced the subjective experience of fluency. Previous research has shown that manipulating fluency can influence truth judgements, with higher truth judgements resulting for high-contrast versus low-contrast images^56^, easy-to-read statements versus difficult-to-read statements^57^, and concrete versus abstract statements^58^ (for a broader overview, see^54^). Therefore, it may be that time pressure interfered with the subjective ease of processing the stimulus (i.e., processing fluency). Alternatively, it may be that not enough information was retrieved from the news headline to elicit a sense of recognition. However, this latter interpretation is at odds with previous literature on the recognition heuristic. Pachur and Hertwig^59^ have shown that the process of retrieving the name of an object (e.g., the name of a city) from memory is very fast and, as a consequence, the effect of recognising an object *increased* under time pressure. In their experiments, the objects recalled from memory (e.g., a city name) were, however, typically less complex than a news headline. Taken together, familiarity seems to be a critical determinant of susceptibility to misinformation, affecting both discrimination ability and response bias. Further research with more robust measures of the process of recognition or repetition under time pressure is encouraged.

How individuals judge online news depends on many factors. We see a need for studies that take a more mechanistic approach and pit competing cognitive accounts against each other (see also^20,60^). For example, evidence accumulation models, including drift-diffusion models^61–63^ (DDM), can account for both choice and response-time data. The DDM approach has been used to examine many decision processes, such as the speed–accuracy trade-off and changes in response tendencies^62^. DDMs could be used to gain a more process-based perspective on the temporal trajectory of discrimination ability and response bias, including how the various determinants influence veracity judgements. For example, DDMs could be used to further explore the role of familiarity: Are familiarity judgements about news headlines instantaneous or are they better explained by a process that unfolds over time? Current interventions to counter misinformation could benefit from such an approach^63^. Ultimately, using insights from more process-based approaches will make it possible to create better-informed, individually-tailored interventions. Such interventions could improve decision making by targeting specific individual characteristics, for example, by informing people about their partisan bias (see also^64^).

Several limitations of our study warrant consideration. Using an external deadline (i.e., a countdown timer) to operationalise social acceleration arguably lacks ecological validity, as there are no response time windows on social media platforms. It would be possible to introduce time pressure by varying the overall time budget of participants in different conditions and leaving it to them to manage the speed–accuracy trade-off across trials. This approach comes with its own set of problems, however: Because people may run out of time towards the end of the experiment, decision times may vary greatly across trials, making the modelling of such processes non-trivial. Another limitation of our study that applies to many misinformation studies relates to the stimuli used. In our study, news headlines were presented as they would appear on Facebook, with an image, a headline, a byline, and a source. In comparison to the less complex stimuli traditionally used for SDT, this set-up introduces variability in how information is processed across participants. For example, the source may play a role in whether news is judged as accurate by some participants but not others. Under time pressure (or generally), participants may have used a source-related heuristic to distinguish true news from false news; we did not explicitly model such potential processes. Future research could use less complex (albeit less ecologically-valid) stimuli, such as the Misinformation Susceptibility Test^65^ (MIST).

To conclude, we experimentally examined the effects of accelerated online dynamics, as operationalised via time pressure, on people’s capacity to identify misinformation. Our results highlight the dangers of rapid news consumption online: Errors in judging the veracity of news are likely to hinder the formation of accurate beliefs. Moreover, prominent drivers of susceptibility to misinformation (e.g., partisan bias and familiarity) remain present under time pressure. A promising avenue for combating online misinformation may thus be to develop interventions aimed at prompting online users to engage in more deliberate thinking.

## Supplementary Information

Supplementary Information 1.

## Data Availability

All information required to replicate our experiment, including the methods and analyses can be freely accessed via the project OSF page (https://osf.io/ag48d/).
