# Supplementary material for: Time pressure reduces misinformation discrimination ability but does not alter response bias
Source: Sci Rep. 2022 Dec 27;12:22416. doi: 10.1038/s41598-022-26209-8 (PMC9794823; doi:10.1038/s41598-022-26209-8)
Supplement: Supplementary file 1 — Supplementary Information 1. [file 41598_2022_26209_MOESM1_ESM.pdf]

Time Pressure Reduces Misinformation Discrimination Ability But Does Not Alter Response Bias

Mubashir Sultan[1,2,*], Alan N. Tump[1,3], Michael Geers[1,2], Philipp Lorenz-Spreen[1], Stefan M. Herzog[1], and Ralf H. J. M. Kurvers[1,3]

[1]Center for Adaptive Rationality, Max Planck Institute for Human Development, Berlin, Germany

[2]Department of Psychology, Humboldt University of Berlin, Germany

[3]Exzellenzcluster Science of Intelligence, Technical University of Berlin, Germany

Supplementary Information

*mubashirsultan@outlook.com

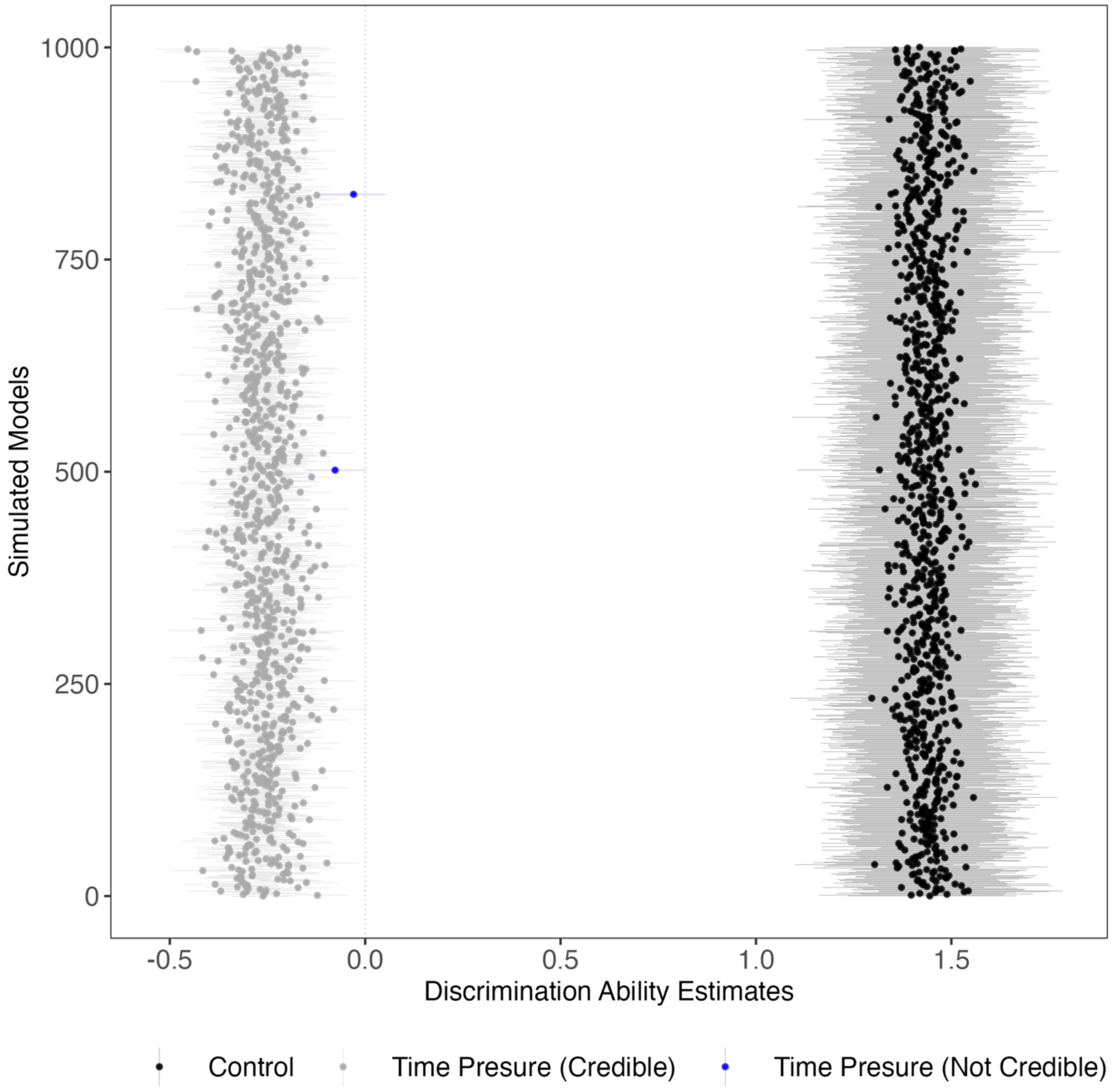

Figure S1. Post-hoc power analysis results for discrimination ability using 1,000 simulated datasets based on our model's posterior. Each dataset was fitted to the same regression model (four chains with 2,000 samples per chain and with 1,000 samples burn-in; e.g., Kurz, 2021). Credibility, like in the main analysis, is assumed if the 95% credible intervals do not include zero. To illustrate, over 1,000 runs of the model, the effect for discrimination ability in the control condition was found 100% of the time (i.e., true positives), and discrimination ability was credibly lower in the time pressure condition in 99.8% of the models. Please note that the effect estimates visualised for the time pressure treatment reflect the difference in the effect as compared to the control condition and not the absolute estimates, which is how they are visualised in the manuscript.

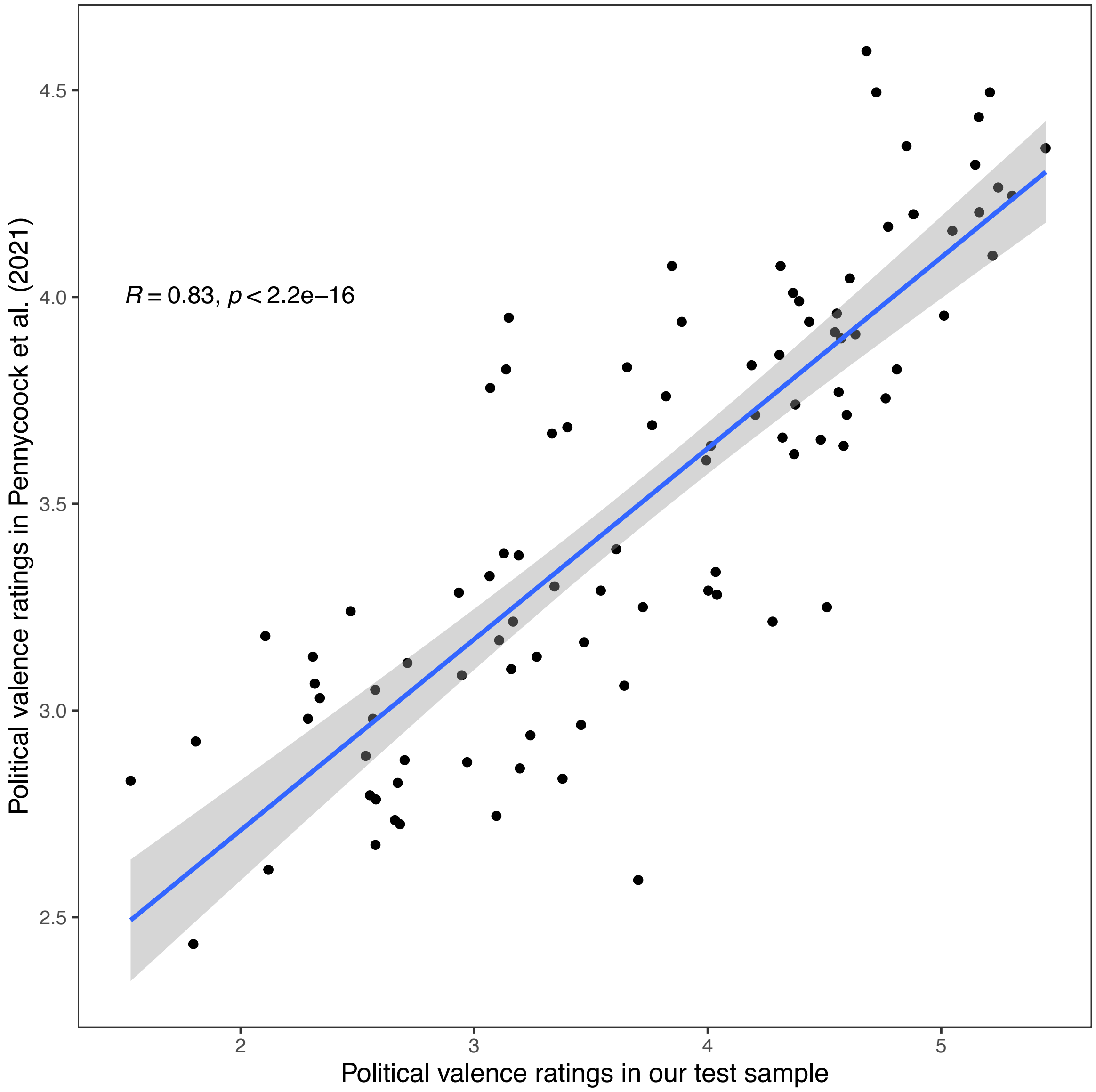

Figure S2. Spearman's rank correlation was computed to compare the political valence ratings of the initial selection of 96 headlines in our test sample and in Pennycook et al. (2021). Results showed a strong positive association, $r_S = .83$, $p < .001$

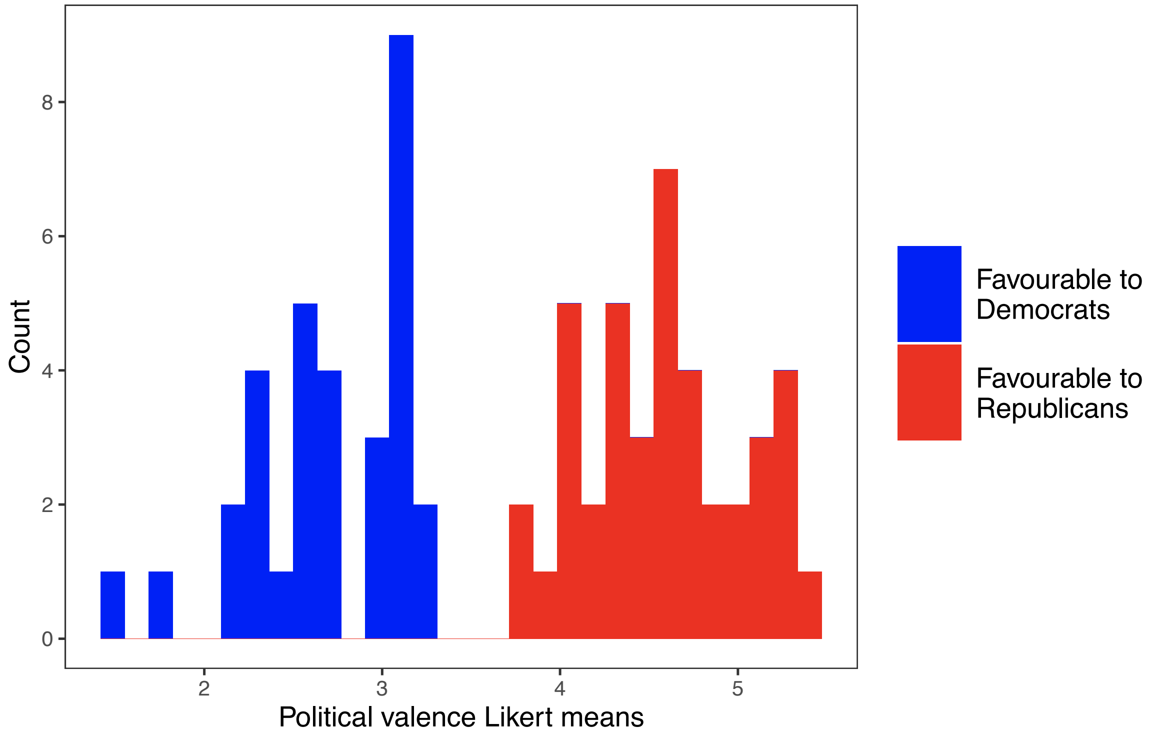

Figure S3. Histogram of item-level political valence means showing a clear demarcation between news headlines favourable to Democrats and Republicans (values between 3.2 and 3.8 were excluded).

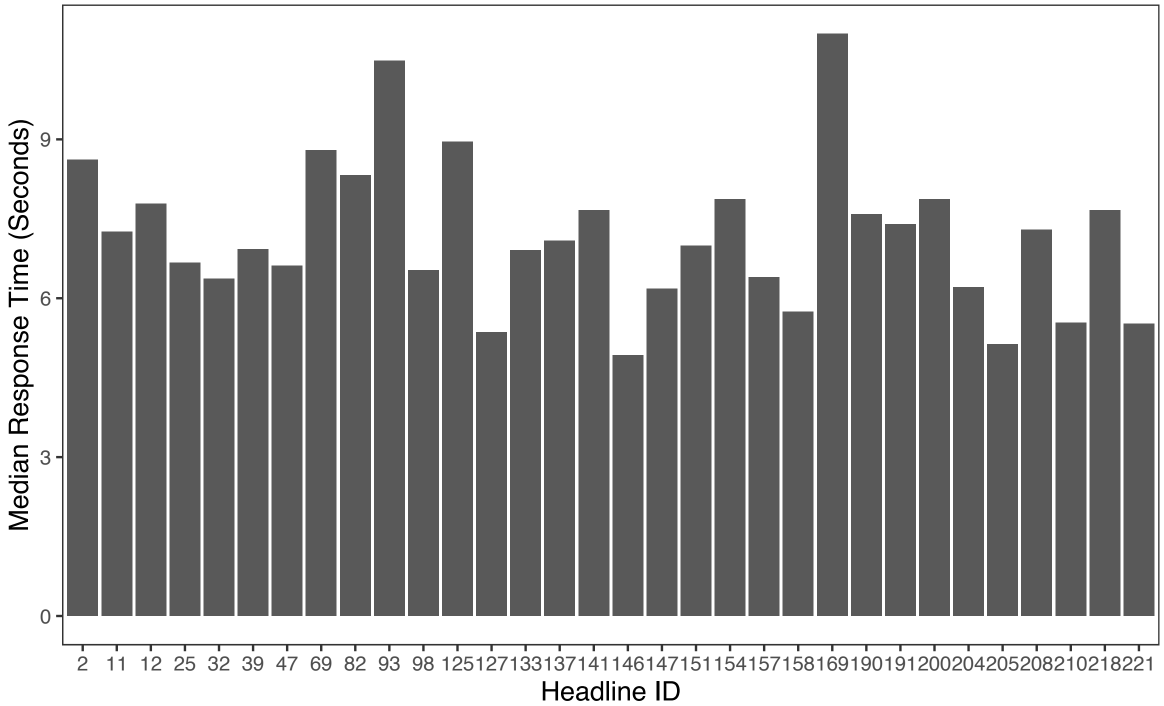

Figure S4. Median response times per headline for the veracity question from the pilot data ($N_{participants}$ = 55). Visual inspection shows that response times were generally around the 6–7 second mark (median across all trials = 6.9). Please note that Headline ID here refers to the Headline ID as enumerated in Pennycook et al. (2021).

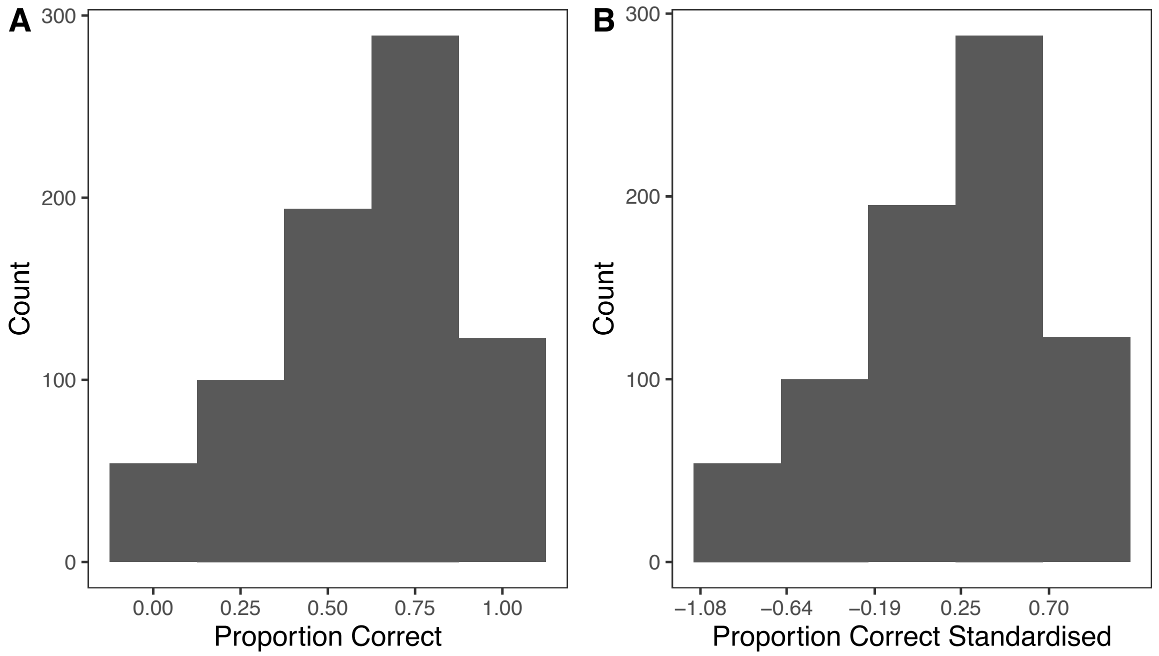

Figure S5. Histogram of (A) non-standardised and (B) standardised proportion of correct CRT responses. Standardisation was achieved by mean centering and dividing by two standard deviations (Gelman, 2008).

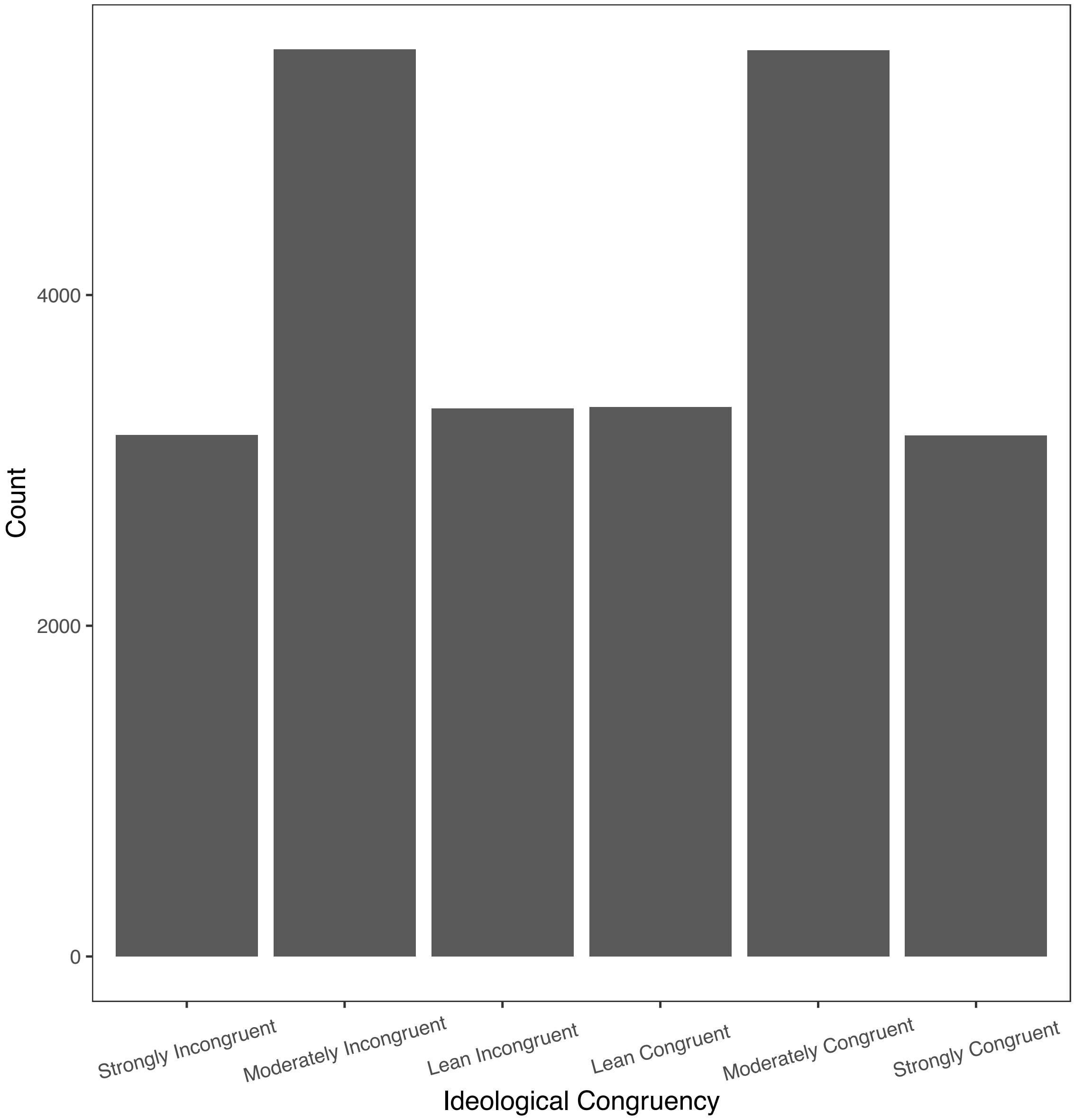

Figure S6. Histogram of non-standardised ideological congruency measure.

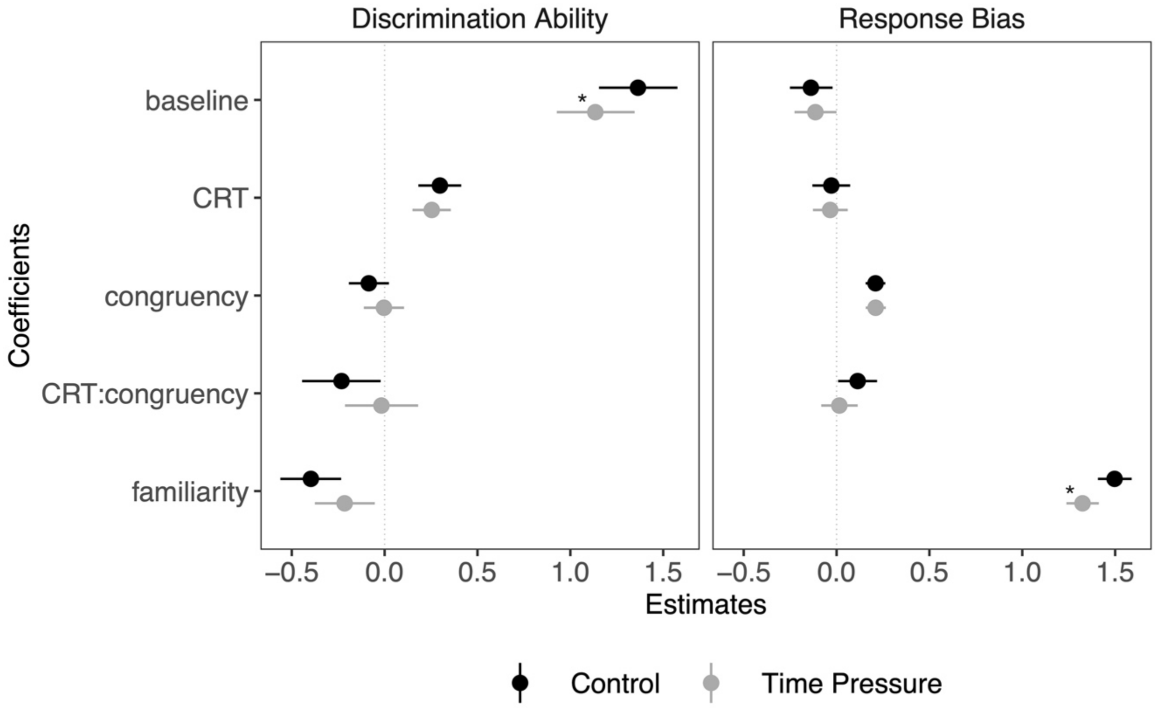

Figure S7. Visualisation of regression coefficients for the control treatment (in black) and the time-pressure treatment (in grey), but with familiarity also mean centered. All results derive from a single SDT analysis using participants' responses (false news or true news) as the response variable, but are shown in two panels to ease interpretation. The left panel shows all estimates for discrimination ability, with more positive (negative) values indicating higher (lower) discrimination ability. The right panel shows all estimates for response bias, with more positive (negative) values indicating a higher likelihood to judge headlines as true (false). Baseline: Overall estimate of discrimination ability (left panel) and response bias (right panel). CRT = Cognitive Reflection Task. Congruency = ideological congruency. Dots represent the mean; error bars represent the 95% CI of the posterior distribution. Credibly different effects between the control and time pressure treatments are marked with an asterisk ('*'). Note that all effects were mean centered except treatment (reference: control).

Table S1
Post-hoc power analysis using 1,000 simulated datasets based on our model's posterior. Each dataset was fitted to the same regression model (four chains with 2,000 samples per chain and with 1,000 samples burn-in; e.g., Kurz, 2021).

		Post-Hoc Power (%)	
Predictor		Discrimination Ability	Response Bias
Baseline	Control	**100**	**100**
	Time Pressure	**99.80**	25.12
CRT	Control	**98.70**	4.29
	Time Pressure	19.04	1.20
Congruency	Control	39.68	**100**
	Time Pressure	27.72	17.45
CRT:Congruency	Control	**58.23**	56.03
	Time Pressure	35.99	31.51
FamiliarityYes	Control	**95.91**	**100**
	Time Pressure	37.49	**61.52**

Note. Credibility, like in the main analysis, is assumed if the 95% credible intervals do not include zero. **Bold values** highlight whether the effect was credible in the main analysis, and thus, reflects the true-positive rate; the non-bold values reflect false-positive rates. Time pressure effects reflect credible differences as compared to the control condition.

Table S2
GLMM Model Interpretation Guide

Reference	Description
Outcome variable	Participant response of whether a news headline is true or false. (Levels: false, true)
Headline veracity	Whether a headline is actually true or false. (Levels: false, true).
Analytical thinking	Proportion of correct scores on the CRT, ranging from 0 to 1.
Ideological congruency	Whether the political leaning of the headline (favourable to Republicans or Democrats) matches the participant's political identity (Strongly Republican to Strongly Democratic). (Levels: Strongly incongruent, Moderately incongruent, Lean incongruent, Lean congruent, Moderately congruent, Strongly congruent).
CRT x ideological congruency	Interaction between analytical thinking and ideological congruency on response bias reflects motivated reflection.
Familiarity	Whether a news headline is reported to be familiar or unfamiliar. (Levels: unfamiliar, familiar).
Discrimination ability	Coefficient estimate for headline veracity (false news, true news) on response variable (either false news or true news). A positive estimate indicates an ability to identify true news as true and false news as false.
Influence on discrimination ability	The estimates of the interactions between headline veracity and the predictors indicate the influence of the respective predictor on discrimination ability.
Model intercept	Response bias.
Influence on response bias	Coefficient estimates of this regression, except those including headline veracity, describe the overall likelihood to classify a given headline as true, and hence indicate the influence of the predictor on response bias.
Time pressure	The grouping variable time treatment describes the influence of time pressure on response bias; the interaction between headline veracity and time treatment describes the influence of time pressure on discrimination ability. (Levels: control, time pressure).

Note. All predictors except time treatment and familiarity were mean centered (i.e., value - mean). CRT and ideological congruency were also divided by two standard deviations after mean centering (Gelman, 2008).

References

Gelman, A. (2008). Scaling regression inputs by dividing by two standard deviations. *Statistics in Medicine*, 27(15), 2865–2873. https://doi.org/10.1002/sim.3107

Kurz, S. A. (2021, April 26). *Bayesian power analysis: Part I. Prepare to reject H_0 with simulation.* A. Solomon Kurz. https://solomonkurz.netlify.app/post/bayesian-power-analysis-part-i/

Pennycook, G., Binnendyk, J., Newton, C., & Rand, D. G. (2021). A Practical Guide to Doing Behavioral Research on Fake News and Misinformation. *Collabra: Psychology*, 7(1). https://doi.org/10.1525/collabra.25293
